# AGE-FRIENDLY MEDITERRANEAN COOKBOOK:CULTIVATING CULTURAL TRANSFORMATION

**DOI:** 10.1093/geroni/igae098.1757

**Published:** 2024-12-31

**Authors:** Sigal Naim, Orit Hirsch-Matsioulas, Ruth Katz, Adi Sapir, Yael Hochman, Anna Zisberg

**Affiliations:** University of Haifa, Haifa, Hefa, Israel; University of Haifa, Haifa, Hefa, Israel; University of Haifa, Haifa, Hefa, Israel; University of Haifa, Haifa, Hefa, Israel; University of Haifa, Haifa, Hefa, Israel; University of Haifa, Haifa, Hefa, Israel

## Abstract

Creating a new reality for old persons on campus, with limited resources but boundless ambition, is both a challenge and an opportunity. In this presentation, we will focus on our experiences over the course of a single academic year, culminating in our university’s inclusion in the global network of Age-Friendly Universities (AFU) – a milestone marking the first institution in the Middle East to achieve such recognition, and only the third in the Mediterranean basin. By embracing the principles of the AFU initiative, we tapped into a broader narrative of societal transformation aligned with the United Nations Sustainable Development Goals (SDGs). Central to our success were cultural values deeply integrated into Israeli society, including collectivity, solidarity, social ties, and audacity. Drawing on these values, we mobilized internal and external stakeholders, forged partnerships, and positioned ourselves as catalysts for change. We conducted comprehensive studies, mapped existing resources, and developed strategic future plans. Our experience illustrates how organizational culture, intertwined with societal dynamics, can drive meaningful change. Community engagement and partnerships played a pivotal role in highlighting the collaborative nature of our initiatives and the importance of involving diverse stakeholders. We invite participants to explore the intersection of culture, age-friendliness, and sustainable development, emphasizing the role of cultural values in shaping age-friendly initiatives. This presentation aims to inspire others to embark on similar journeys, utilizing culture to create inclusive environments where older adults can thrive.

